# Feasibility of C-reactive protein point-of-care testing for antibiotic stewardship in rural GP–pharmacy settings

**DOI:** 10.1017/ash.2026.10401

**Published:** 2026-05-20

**Authors:** Sajal Kumar Saha, Michael Muleme, Oliver Van Hecke, Mary Lou Chatterton, Eugene Athan

**Affiliations:** 1 Centre for Innovation in Infectious Disease and Immunology Research (CIIDIR), Institute for Mental and Physical Health and Clinical Translation (IMPACT), School of Medicine, Deakin University, Geelong, VIC, Australia; 2 Barwon Southwest Public Health Unit, Geelong, VIC, Australia; 3 Department of Public Health and Primary Care, Ghent University, Ghent, Belgium; 4 Monash University Health Economics Group, School of Public Health and Preventive Medicine, Monash University, Melbourne, VIC, Australia

## Abstract

**Objective:**

To assess the feasibility of implementing C-reactive protein point-of-care testing (CRP-PoCT) for patients presenting with respiratory tract infections (RTIs) in primary care.

**Design:**

Feasibility study.

**Setting:**

General practice (GP) and community pharmacy in rural Victoria.

**Patients (or Participants):**

Five GPs and a community pharmacist used CRP-PoCT for RTI patients aged 12–65 years for two months between August and December 2023.

**Methods:**

Data on testing, prescribing, and implementation barriers and facilitators were collected from patient records and surveys of GPs and pharmacists. Feasibility outcomes were analyzed using descriptive statistics and content analysis.

**Results:**

Fifty-five patients were screened, with recruitment rates of 87.5% (35/40) in GP clinics and 67% (10/15) in community pharmacies, and a retention rate of 100%. Adherence to the testing protocol was 100% in both settings; however, adherence to the prescribing protocol was 76% in GP practices and 100% in pharmacies. Common RTI symptoms were cough (70%, n = 30), fever (58%, n = 25), and sore throat (44%, n = 19). Antibiotics were prescribed to 36.4% (12/33) of GP patients and 10% (1/10) of pharmacy patients. Pharmacists managed patients with OTC medicines (80%, 8/10) or immediate GP referral (10%, 1/10). CRP-PoCT feasibility was rated highly by GPs [median 6/7 (IQR 4–6)] and moderately by the pharmacist (4/7). Key implementation facilitators identified were nurse/technician support, medicare funding, clear workflows, and GP–pharmacy collaborative practice agreement.

**Conclusion:**

With mitigation of practical challenges, CRP-PoCT can be feasibly implemented in rural GP–pharmacy settings to enhance antibiotic stewardship for acute respiratory infections in primary care.

## Introduction

Over 80% of human antibiotic consumption occurs in the community, mostly for acute respiratory infections.^1^ Antibiotic resistance substantially increases patients’ illness burden in the community,^2^ and minimizing antibiotic use reduces the emergence of resistant infections.^3^ The tools that leverage identifying patients who have self-limiting respiratory tract infections (RTIs) can reduce unnecessary use of antibiotics. C-reactive protein (CRP), a biomarker of inflammation, can be such a tool for use by GPs and community pharmacists to improve antibiotic stewardship in RTIs. ^4,5^

Cochrane reviews^6^ of 11 cluster randomized controlled trials show that CRP point-of-care testing (CRP-PoCT) reduces antibiotic use by 24% in adults with RTIs (RR 0.76; 95% CI 0.68–0.86). A 2025 systematic review^7^ of 33 studies found that CRP and Group A Streptococcus point-of-care tests are the most commonly used diagnostics by GPs and community pharmacists in RTI management. However, CRP-PoCT’s implementation feasibility and scalability remain poorly understood in rural GP–pharmacy settings.^8^ It is proven that GP–pharmacist team-based stewardship strategies can reduce GPs’ antibiotic prescribing by 12% and improve guideline-adherent prescribing by 16%.^9^ To date, globally, the opportunity to implement CRP-PoCT using a GP–pharmacy collaborative care model remains less explored, except for a few developed countries like the UK, Ireland, Canada, and USA.

In 2024, the Australian Commission reported a 25% reduction in antimicrobial use from 2019 to 2021 in the community under the Antimicrobial Use and Resistance in Australia surveillance program.^10^ Despite this decline, community antibiotic dispensing in Australia remains substantially higher than in countries such as the Netherlands and Scandinavia.^11^ In particular, GPs’ RTI-related antibiotic prescribing remains much higher than recommended benchmarks, such as for acute bronchitis/bronchiolitis (72%).^12^ Victorian GPs use antibiotics for 67% of sore throat cases and often choose broad-spectrum antibiotics in more than 47% of cases. These prescribing rates are higher among children and older age groups and are inconsistent with the Australian Clinical Practice guidelines (eTG Antibiotic).^13^ Pharmacists currently rely on symptom-based recommendations to manage RTIs. Incorporating CRP-PoCT in GP and pharmacy settings may reduce unnecessary GP visits and delays in timely consultation, support appropriate referrals, and optimise antibiotic use.^14,15^

Although CRP-PoCT has demonstrated feasibility in urban community pharmacies in Western Australia,^16^ evidence is limited in the context of rural pharmacy and GP–pharmacy co-located models across Australia. This is particularly important in rural Australia, where patients face long travel distances, limited access to GPs, and greater distances to hospital services. The experiences and attitudes of the CRP-PoCT users can play a critical role in the design of a GP–pharmacist interprofessional care model for implementing CRP-PoCT. This study aimed to assess the feasibility of implementing CRP-PoCT in closely located rural GP–pharmacy practices in Victoria, Australia.

## Methods

This feasibility study was conducted in closely located GP and community pharmacy practices (400 m apart) in a rural town near Geelong, Victoria, Australia, between August and December 2023. Feasibility outcomes were reported using the framework by Leon, Davis, and Kraemer (2011).^17^

### Implementation of the CRP-PoCT program

CRP-PoCT analyzers (Afinion 2, Abbott, QLD, Australia) were installed in the pharmacy (Aug–Sep 2023) and GP clinic (Oct–Dec 2023). Five GPs and one pharmacist were trained by the investigator (SKS) and an Abbott trainer. Clinical algorithms (Diagrams 1–2, Supplementary Appendix) were developed using international evidence and a previous Western Australian study,^16^ and validated by academic pharmacists and an infectious diseases physician. The algorithm guided CRP-PoCT use and antibiotic prescribing.

Patients aged 12–65 years with RTI symptoms attended the pharmacy between August and September 2023. The pharmacist screened for patient eligibility, obtained patient consent, and performed clinical assessment, and CRP-PoCT. Based on CRP results and clinical judgment, patients received treatment advice and GP referrals as needed. The pharmacist consulted paired GPs for antibiotic scripts, counseled patients on results and recommendations, and followed up by phone on day 3 for further management or referral.

Patients aged 12–65 years presenting to the GP clinic between October and November 2023 were assessed for eligibility for CRP-PoCT, excluding those positive for COVID-19 or influenza. After providing written consent, patients were tested in the nursing area and returned to the GP with results for treatment decisions. GPs and pharmacists used CRP values alongside clinical judgment according to the protocol to recommend antibiotic use. Neither clinic nor pharmacy was blinded, and referrals between settings were allowed during the study. Potential participants who declined the test use received usual care.

### Inclusion and exclusion criteria

**Inclusion:** Patients with age 12–65 years, acute cough < 4 weeks or ≥1 symptom (shortness of breath, wheeze, chest pain, fever, perspiring, headache, myalgia, feeling unwell).

**Exclusion:** Patients with chronic inflammatory disease on immunosuppressants, pregnant/breastfeeding, severe symptoms needing immediate GP referral, or using long-term antibiotics.

### Sample size

The minimum target was 40 tests in the GP clinic and 15 in the pharmacy over two months, without any financial incentives. These targets were pragmatically selected, taking into account the anticipated decline in RTI consultation rates later in winter and prior Australian CRP PoCT pharmacy studies, which recruited 7–30 patients per pharmacy over ∼8 weeks during winter.^17^

### Survey of GPs and pharmacist

Six GPs and a pharmacist were eligible and completed a voluntary survey assessing CRP-PoCT’s feasibility, clinical value, impact on antibiotic use, and attitudes towards implementing CRP-PoCT through GP–pharmacist collaboration. Responses used Yes/No and 1–5 Likert scales. Survey included open-ended qualitative questions to capture the data related to barriers and facilitators of the testing. Survey completion implied consent.

### Outcomes

Feasibility outcomes: rate of recruitment, retention, and adherence to protocol. Clinical outcomes: a) Number of CRP tests conducted in GP and pharmacy practices and b). Rate of antibiotic prescription among patients tested. Operational outcomes: a) Views and experiences of GPs and pharmacists on the implementation feasibility of the testing, b) attitudes toward future use and GP–pharmacist collaborative implementation of the testing, and c) barriers and facilitators of using the testing in routine patient care.

### Data collection and analysis

Data on testing, diagnosis, and prescribing were collected from patients’ medical records using Best Practice software. Data reflecting operational feasibility outcomes were collected via postimplementation surveys of participant GPs and a pharmacist. Descriptive statistics summarized participant demographics, test use, and antibiotic prescribing. Survey responses were analyzed using the median and interquartile range (IQR). Analyses were conducted using SPSS version 25 (IBM, Armonk, United States of America). Free-text responses on barriers and facilitators to test use were reported descriptively.

### Ethics

Ethics approval was obtained from the Barwon Health Human Research Ethics Committee on July 27, 2023 (Review Reference HREC/94724/VICBH-2023-383230 [v3]) and the Deakin University Human Research Ethics Committee on September 11, 2023 (Ref 2023-269).

## Results

### Recruitment, retention, and adherence rate

Table 1 describes the feasibility constructs. A total of 55 patients were screened over eight weeks, with recruitment rates of 87.5% (35/40) in general practice (GP) and 67% (10/15) in pharmacies. Weekly recruitment ranged from 2–10 participants in GP and 0–3 in pharmacies. Retention was 100% when defined by completion of required study data; however, pharmacist follow-up was achieved in 40% of cases. Reasons for non-recruitment after screening in GP clinics included patient unwillingness, ongoing antibiotic treatment, and need for hospital referral, while in pharmacies, they included limited time, patient unwillingness, and need for GP referral (Table 1). Adherence to the testing protocol was 100% in both settings, while adherence to the prescribing protocol was 76% in GP practices and 100% in pharmacies.

**Table 1.** Feasibility of implementing CRP-PoCT in rural GP clinic and community pharmacy

Construct	GP Clinic	Pharmacy
Screening (Patients screened)	40	15
Reasons for exclusion	5 excluded 1 unwilling to take part 2 GPs’ feeling of unlikely need for antibiotics 1 was on antibiotic use 1 referral to the hospital	5 excluded 1 -patient denial 1-time poor patient 2-GP referral 1-pharmacist’s time shortage
Recruitment	35 (87.5%)	10 (67%)
Randomization (patients eligible for testing)	35 (87.5%)	10 (67%)
Invalid CRP result	2 (0.05%)	0 (0%)
Valid CRP result	33 (94.2%)	10 (100%)
Retention		
Provided the required data	33 (100%)	10 (100%)
Patients follow-up	Not assessed	4 (40%)
Adherence to testing protocol	33 (100%)	10 (100%)
Adherence to treatment (prescribing) protocol	26/33 (78.8%)	10 (100%)

### Demographic characteristics and presenting symptoms

The demographics of patients and presenting symptoms are reported in Table 2. The median age of patients was 49 years (interquartile range [IQR]: 41–61). Of the patients, 22 (51%) were aged 19–49 years, while 21 (49%) were aged 50 years and above. The top five signs and symptoms were cough (70%, n = 30), fever (58%, n = 25), sore throat (44%, n = 19), runny nose (39%, n = 17), and headache (37%, n = 16). Out of 33 patients tested with valid CRP results in GP clinics, five patients (15%) had penicillin allergy, and one for doxycycline allergy.

**Table 2.** Participant’s demographics, clinical symptoms, and antibiotic prescriptions. (IQR: interquartile range)

Variable			
Gender (female)	23 (53.4%)	–	–
Age [median (IQR)]	49 years [IQR: 41–61Years]		
Penicillin allergy	15% (5/33)	–	–
Symptoms	N (%)	Antibiotics prescribed N = 13 (%)	No antibiotics prescribed N = 30 (%)
Cough	30 (70)	10 (77)	20 (67)
Fever	25 (58)	11 (85)	14 (47)
Sore throat	19 (44)	7 (54)	12 (40)
Runny nose	17 (39)	5 (38)	12 (40)
Headache	16 (37)	3 (23)	13 (43)
Congestion	11 (26)	2 (15)	9 (30)
Cold (chilling)	10 (23)	3 (23)	7 (23)
Phlegm	9 (21)	3 (23)	6 (20)
Sinusitis	6 (14)	3 (23)	3 (10)
Asthma	5 (12)	3 (23)	2 (6.7)
Sweating	4 (9)	1 (7.7)	3 (10)
Upper respiratory tract infection	4 (9)	1 (7.7)	3 (10)
Vomiting	4 (9)	2 (15)	2 (6.7)
Pneumonia	3 (7)	2 (15)	1 (3.3)
Diarrhea	3 (7)	1 (7.7)	2 (6.7)
Bronchitis	3 (7)	1 (7.7)	2 (6.7)
Hoarse voice	3 (7)	2 (15)	1 (3.3)
Dizziness	3 (7)	0 (0)	3 (10)
Sputum color	3 (7)	1 (7.7)	2 (6.7)
Vertigo	1 (2)	0 (0)	1 (3.3)
Muscle pain	1 (2)	0 (0)	1 (3.3)

## Clinical outcomes

### CRP test results

Table 3 and 4 show CRP results by antibiotic prescriptions and clinical symptoms respectively. For patients in the GP clinic, CRP ranged from <5 to 177 mg/L: 0–20 mg/L in 70% (n = 23), 21–40 mg/L in 15% (n = 5), 41–100 mg/L in 12% (n = 4), and >100 mg/L in 3% (n = 1). No adverse outcomes were reported, though two invalid results required researcher’s follow-up.

**Table 3.** CRP levels and antibiotic prescriptions

Settings			CRP level			
GP		Total	0–20 mg/L	21–40 mg/L	41–100mg/L	>100 mg/L
	Patient tested	33	23 (70%)	5 (15%)	4 (12%)	1 (3%)
	Proportion prescribed antibiotic	36.36% (12/33)	21.7% (5/23)	40% (2/5)	100% (4/4)	100% (1/1)
	Prescribed antibiotic	Amoxicillin 7 (58%) Doxycycline 3 (25%) Amoxicillin and doxycycline 1 (8%)				
Pharmacy	Patient tested	10	7	1	1	1
	Proportion recommended antibiotic prescription in consultation with a GP	10% (1/10)	0	0	0	100% (1/1)
	Referred to GP after testing	20% (2/10)	0	0	100% (1/1)	100% (1/1)
	Prescribed antibiotic	Amoxicillin (1)	–	–	–	–

For patients in the pharmacy, CRP ranged from <5 to 110 mg/L: 0–20 mg/L in 70% (n = 7), 21–100 mg/L in 20% (n = 2), and >100 mg/L in 10% (n = 1). Based on CRP and routine assessment, 80% (8/10) received OTC medicine. Pharmacists provided self-care advice to all patients, including rest (90%), hydration (80%), and GP follow-up if symptoms persisted (20%). One patient was referred to a GP immediately. No adverse outcomes or issues related to the testing device were reported.

The median CRP among all participants was 9 mg/L (IQR: 5–23). Although median CRP was higher in those prescribed antibiotics (35 mg/L; IQR: 10–60) than in those not prescribed (7 mg/L; IQR: 5–12) (Supplementary Tables 2–3), GPs still prescribed antibiotics for patients with CRP levels below recommended thresholds (Table 3).

### Antibiotic prescribing

Antibiotics were prescribed to 30.2% (13/43) of participants with valid CRP results across GP and pharmacy settings (Table 3). In general practice, 36.4% (12/33) received antibiotics, mostly amoxicillin (7, 58%) or doxycycline (3, 25%), with one patient receiving both. Pharmacists prescribed one antibiotic (amoxicillin) in consultation with a GP (10%, 1/10). Most patients had CRP <20 mg/L—70% in GP clinics and 70% in pharmacies—indicating no need for antibiotics. Around 22% with CRP level 0–20 mg/L received antibiotic prescriptions from GPs against the study protocol. 20–30% with CRP 21–100 mg/L suggested delayed or no prescribing, and <3% had CRP >100 mg/L who received antibiotics.

## Feasibility outcomes

### Implementation feasibility of CRP-PoCT in GP and pharmacy settings

Appraisal of CRP-PoCT strategies indicated high feasibility among GPs (median 6/7; Table 5) and moderate feasibility among pharmacists (4/7; Supplementary Table 4). Most GPs (>83%) rated the service as relevant, affordable, effective, and safe, with potential to improve patient care. Pharmacists agreed on relevance, effectiveness, and safety, but were less confident about ease of delivery, staff acceptability, and affordability. GPs considered delayed prescribing based on CRP feasible, whereas pharmacists were less certain. Both groups viewed explaining CRP results and engaging patients as highly feasible. Overall, collaborative implementation was seen as relevant, safe, and effective, with feasibility around 5.5/7.

**Table 4.** Clinical symptoms and CRP value

Symptoms	20–100+ mg/L N = 14 (%)	<20 mg/L N = 29 (%)
Sore throat	9 (64)	10 (34)
Fever	9 (64)	16 (55)
Cough	8 (57)	22 (76)
Runny nose	5 (36)	12 (41)
Headache	5 (36)	11 (38)
Congestion	3 (21)	8 (28)
Cold (chilling)	3 (21)	7 (24)
Phlegm	3 (21)	6 (21)
Pneumonia	2 (14)	1 (3.4)
Bronchitis	2 (14)	1 (3.4)
Sinusitis	2 (14)	4 (14)
Hoarse voice	1 (7.1)	2 (6.9)
Asthma	1 (7.1)	4 (14)
Vomiting	1 (7.1)	3 (10)
Sputum color	1 (7.1)	2 (6.9)
Upper respiratory tract infection	1 (7.1)	3 (10)
Sweating	0 (0)	4 (14)
Vertigo	0 (0)	1 (3.4)
Diarrhea	0 (0)	3 (10)
Muscle pain	0 (0)	1 (3.4)
Dizziness	0 (0)	3 (10)

**Table 5.** GPs appraisal and implementation feasibility of the CRP-PoCT program for antibiotic stewardship in respiratory infections

	POCT CRP testing	Is it Relevant (n/N) (% Yes)	Is it affordable? (n/N) (% Yes)	Can it be delivered easily? (n/N) (% Yes)	Will it be effective at reducing antimicrobial use? (n/N) (% Yes)	Will it be acceptable to staff? (n/N) (% Yes)	Is it safe to implement? (n/N) (% Yes)	Will it improve patient care? (n/N) (% Yes)	Rating of implementation feasibility out of 7 scale [median, (IQR)
1	Using POCT CRP testing service for antibiotic stewardship in RTIs	6/6 (100)	5/6 (83)	5/6 (83)	6/6 (100)	5/6 (83)	6/6 (100)	6/6 (100%)	6 (4–6)
2	Recommending delayed/back-up prescriptions based on CRP result	4/6 (67)	5/5 (100)	5/6 (83)	5/6 (83)	5/6 (83)	6/6 (100)	5/6 (83%)	6 (4–6)
3	Addressing patient demand for antibiotics by explaining CRP value and relevant conversation with patients	5/5 (100)	3/3 (100)	4/5 (80)	5/5 (100)	4/5 (80)	5/5 (100)	5/5 (100%)	6 (6–6)
4	Implementing POCT CRP testing program both in GP and pharmacy practices through GP–pharmacist collaboration where possible	5/5 (100)	3/3 (100)	5/5 (100)	5/5 (100)	5/5 (100)	5/5 (100)	5/5 (100%)	5.5 (4.5–6.5)

Note: Feasibility of PoCT-CRP implementation strategies were measured using Yes/No response. Overall feasibility was measured using scale 1–7 and presented by median and interquartile range (IQR). N = Total number of GPs participated in the survey and n = number of GPs responded to the survey items.

### Patients follow up by pharmacist

Of 10 patients tested, pharmacists successfully followed up with only four patients; six could not be contacted despite multiple attempts. One patient reported no symptom improvement by day three but did not consult a GP or take antibiotics. For all followed-up patients, pharmacists provided ongoing management advice, and none required GP referral.

### Perceived benefits of using CRP-PoCT by GPs and pharmacist

GPs and pharmacists had mixed perceived benefits of using CRP-PoCT and on whether testing was time-consuming but strongly agreed that it supported RTI assessment, guided antibiotic prescribing, increased confidence, and improved patient communication (Table 6 and Supplementary Table 4). However, GPs did not always follow recommended prescribing guidance despite access to CRP-PoCT for instance recommending antibiotics for 24% of patients with CRP < 40 mg/L.

**Table 6.** Perceived benefits of using CRP-PoCT by GPs

No	Statement	Median	IQR
1	POCT CRP testing program was not time consuming (n = 5)	3	2.5–3.5
2	POCT CRP testing helped me to diagnose the severity of RTI infection? (n = 5)	4	4–5
3	POCT CRP testing result helped my decision for antibiotic therapy in RTI patients (n = 5)	4	3–5
4	POCT testing program increased my confidence for RTI management (n = 5)	4	3–4.5
5	POCT testing program increased my conversation with patient to address patient demand for antibiotic (n = 5)	5	4.5–5
6	POCT testing program increased my confidence for delayed antibiotic prescription (n = 5)	4	4–4.5

Note: Belief measured on a scale of 1–5, where 1 = poorly believe and 5 = strongly believe. n = number of participants and IQR, interquartile range.

### Attitudes towards future use of CRP-PoCT testing by GPs and pharmacist

Table 7 and Supplementary Table 4 summarise attitudes toward future CRP-PoCT use. GPs agreed more strongly than pharmacists that they would use CRP-PoCT if available in their practice. Both groups agreed that CRP-PoCT could reduce antibiotic use in RTIs and optimize treatment duration, although GPs were less convinced about optimizing antibiotic choice or reducing broad-spectrum antibiotic (eg, roxithromycin, gentamycin, Cephalexin) use. Both groups strongly supported Medicare coverage for CRP-PoCT (eg, $15–$20). GPs indicated that practice nurses should support testing, while pharmacists preferred training other pharmacy staff.

**Table 7.** GPs attitudes toward CRP-PoCT use and implementation in future

No	Statement	Median	IQR
1	I would like to use POCT CRP testing in the future if available in my practice (n = 5)	4	3–4
2	I would like to refer the RTI-patient to a collaborative community pharmacy for doing the POCT CRP testing if available (n = 5)	4	3–4
3	POCT CRP testing program can be implemented through GP–pharmacy collaborative practice agreement (n = 5)	4	4–4
4	POCT CRP testing program can help efficient patient referral between GPs and community pharmacists if the service is available both in GP and pharmacy practices (n = 5)	4	4–5
5	Practice nurses should support doing POCT testing in my practice (n = 5)	5	4–5
6	POCT CRP testing program would impact general practice by (n = 5)		
	Decreasing overall antibiotic prescribing	4	4–5
	Decreasing broad-spectrum antibiotic prescribing	3.5	3–5
	Optimizing the choice of antibiotics while prescribing	3	2–5
	Optimizing the duration of antibiotic therapy while prescribing	4	2–5
7	Patient Medicare should cover the costs of POCT CRP testing (e.g., $15–20) (n = 5)	5	5–5
8	POCT CRP use could increase GP–pharmacist communication with regard to RTI management (e.g., patient referral and antibiotic use) (n = 5)	5	4–5

Note: Attitudes measured on a scale of 1–5, where 1 = strongly disagree and 5 = strongly agree. n = number of participant GPs and IQR, interquartile range.

### GPs attitudes towards GP–pharmacist collaborative implementation of the CRP-PoCT

GPs and pharmacists strongly agreed that CRP-PoCT could be implemented through a GP–pharmacy collaborative practice agreement (Table 7; Supplementary Table 4). Both groups believed CRP-PoCT would enhance communication regarding RTI management, including referrals and antibiotic use, with both professions anticipating more efficient bidirectional referral (Table 7; Supplementary Table 4).

### Challenges and opportunities of using CRP-PoCT by GPs and pharmacist

Both pharmacist and GPs reported initial challenges with implementing CRP-PoCT into routine care, primarily related to time constraints, competing responsibilities, and the need for staff education. These challenges were exacerbated in pharmacies where a single pharmacist managed all aspects of care. Facilitators identified included upskilling staff, clear workflow protocols, improved communication and collaboration between pharmacists and GPs, increased public awareness of testing and appropriate antibiotic use, pharmacy-based testing prior to GP visits, and funding models to ensure no cost to clinics and improved affordability for patients (supportive quotes, supplementary table 5).

## Discussion

To our knowledge, this is the first study examining the feasibility of implementing CRP-PoCT in rural primary care within integrated GP–pharmacy models in Australia. Meeting recruitment targets, along with good retention and protocol adherence to testing, suggests CRP-PoCT can be integrated into routine practice if practical challenges are addressed. This aligns with international experiences from Denmark,^18–19^ Sweden,^20–23^ and the Netherlands,^24–25^ where CRP-PoCT is routinely used for managing RTIs in GP settings. Both GPs and pharmacist reported positive attitudes and perceived clinical utility regarding CRP-PoCT use. Support from nurses in GP clinics and pharmacy assistants was identified as critical for integration of CRP-PoCT into busy workflows.

In this feasibility study, 63% of tested patients were not prescribed antibiotics, consistent with published reviews and RCTs (32–60%).^26,27^ This highlights the potential impact of CRP-PoCT and the need to establish effect size in reducing antibiotic prescribing in rural Australian primary care in future. However, GPs prescribed antibiotics in 24% of cases despite low CRP levels (<40 mg/L), contrary to the clinical algorithm. Reasons for this remain unclear and warrant further qualitative investigation.

While both GPs and pharmacist expressed confidence in using CRP results, effective patient communication was identified as key to managing expectations and supporting appropriate prescribing, consistent with successful implementation approaches in Nordic settings.^19,21^ Notably, 100% protocol adherence in pharmacies supports the feasibility of opportunistic implementation in this setting. However, we found that pharmacist was able to follow up only 40% of tested patients, with the remainder unreachable despite multiple attempts, likely reflecting patient-related factors.

Participant GPs and pharmacist supported CRP-PoCT implementation using a formal GP–pharmacy collaborative practice agreement. Establishing such agreements in future could enhance interprofessional communication and streamline RTI patient management and referrals in Australian primary care.^28^ Similar model in Northern Ireland^29^ has demonstrated high patient referral rates (60% of 328 patients) in research context from GPs to pharmacies for CRP testing.

While a small proportion of patients were excluded due to pharmacist time constraints (3/15) and invalid testing in GP settings (2/40), these findings highlight important real-world feasibility challenges. Furthermore, only 4/10 patients who were tested in pharmacy were followed up by a pharmacist, while six were unreachable despite multiple contact attempts, a barrier to ensuring reliable patient follow-up. These are important considerations for real-world implementation of CRP-PoCT.

Indeed, implementation of CRP-PoCT required additional time and resources, including staff training and approximately 3–5 minutes for test administration, as well as integration into existing workflows. Therefore, task delegation could be key for workflow adaptation. As study participants reported, if nurses in GP clinics and pharmacy assistants in community pharmacies undertake patient screening, sample collection, and test operation, the test would be highly feasible to use during routine patient care. This team-based approach will likely reduce the time burden on GPs and pharmacists while maintaining clinical oversight for decision-making. The published studies have highlighted the similar implementation barriers such as time constraints, workflow disruption, limited training, low trust in CRP as a non-specific biomarker, concerns about over-reliance, and reduced perceived utility when diagnoses are clinically apparent.^7,17,28^

### Strengths and limitations

To our knowledge, this is the first Australian study demonstrating the feasibility of implementing CRP-PoCT in closely located rural GP–pharmacy settings. This study has several limitations. It was a single-center study and conducted over a short two-month period due to funding constraints, with no baseline data collected as the focus was feasibility rather than effectiveness. The observational design, small sample size, and absence of pediatric participants limit generalizability. Conducted at the end of winter, the study likely captured fewer RTI presentations, resulting in a limited number of eligible patients, as noted by participants. Detailed medical history was not systematically collected, limiting assessment of potential CRP-related confounders. Survey responses were from a small, highly engaged sample and may not reflect broader clinician perspectives. In addition, no data on COVID-19 or influenza testing were collected, and actual service uptake was not measured, limiting insights into real-world implementation. A separate qualitative study is planned to address these gaps.

### Implications and future directions

Overall, the findings suggest that CRP-PoCT could enhance antibiotic stewardship in rural Australian settings, aligned with international best practices. This study provides an insight for future trials, refinement of implementation pathways, scaling up, and optimisation of the CRP-PoCT service in rural GP-community pharmacy settings in Australia. Broader adoption of the testing in this setting will require incentives, training, clear guidelines, regulation, governance, and policy support.^5,7,28–30^ Participants highlighted Medicare support, nurse assistance, and national policies as key facilitators, consistent with international experience.

Future innovative trials can be designed to assess the impact of pharmacist involvement and use of the testing on GPs antibiotic prescribing in RTIs. The evidence effectiveness and cost-effectiveness is central for policy impact. Future assessment for CRP-PoCT could be extended for GP-pharmacy practices which are co-located vs not co-located and identify the relevant barriers and facilitators.

## Conclusion

With mitigation of practical challenges, CRP-PoCT can be feasibly implemented in rural GP–pharmacy settings to enhance antibiotic stewardship for acute respiratory infections in primary care. A rural GP–pharmacy collaborative care model for implementing CRP testing could be an opportunity to improve antibiotic stewardship in future.

## Supporting information

10.1017/ash.2026.10401.sm001 Saha et al. supplementary material Saha et al. supplementary material

## Data Availability

Supporting data is available on request and after permission from the ethics committee.

## Supplementary material

The supplementary material for this article can be found at https://doi.org/10.1017/ash.2026.10401
