# Supplementary material for: Feasibility of C-reactive protein point-of-care testing for antibiotic stewardship in rural GP–pharmacy settings
Source: Antimicrob Steward Healthc Epidemiol. 2026 May 20;6(1):e148. doi: 10.1017/ash.2026.10401 (PMC13199417; doi:10.1017/ash.2026.10401)
Supplement: Saha et al. supplementary material [file S2732494X2610401Xsup001.docx]

**Supplementary Appendix A**

**Diagram 1: The algorithm (UK guideline) followed by GPs for clinical assessment and use of the CRP-PoCT** [Adapted from Primary Care Respiratory Update 2022 guidance. <https://www.pcrs-uk.org/sites/default/files/PoC%20Testing%20FINAL3.pdf>]

**Diagram 1A.** Algorithm for RTIs that are not associated with COPD


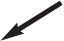


+ve COVID-19

+ve Influenza

**-ve COVID-19**

**and Influenza**

If prescriber feels antibiotics are required carry out **CRP-POCT** using finger prick blood sample

**CRP <20mg/l**

**CRP 20-100mg/l**

**Patient presents with RTIs**

**Prescribe antibiotics**

**Consider delayed antibiotic prescription (for use at later date if symptoms worsen)**

**Antibiotics unlikely to be beneficial - reassurance and symptomatic relief**

**CRP >100mg/l**

**Influenza pathway**

**COVID-19**

**pathway**

Optional tests

**COVID-19 test Influenza test**

**Triage -** Clinical Decision Rules (see table 1)

**Diagram 1B.** Algorithm for IE-COPD


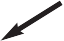


**Triage -** Clinical Decision Rules (see table 1)

**-ve COVID-19 and Influenza**

If prescriber feels antibiotics are required carry out **CRP-POCT** using finger prick blood sample

**CRP <20mg/l**

**CRP 20-40mg/l**

**Patient presents with RTIs**

**Prescribe antibiotics**

**Antibiotics may be beneficial if purulent sputum present**

**Antibiotics unlikely to be beneficial - reassurance and symptomatic relief**

**CRP >40mg/l**

**Influenza pathway**

**COVID-19**

**pathway**

+ve Influenza

+ve COVID-19

Optional Tests

**COVID-19 test**

**Influenza test**

| **Table 1: Clinical signs and symptoms that indicate possible bacterial causes of RTI or IE-COPD** |
| --- |
| Chest crackles: In people with raised temperature, chest crackles could indicate the need for antibiotics irrespective of CRP levels |
| Cough: prescribers should exclude post-infective cough |
| History of recent antibiotic use |
| Increased breathlessness |
| Oxygen saturation <95% |
| Possible fever (≥38oC); viral infections are more likely than bacterial pathogens to cause a fever |
| Raised heart rate >100 beats per minute |
| Raised respiratory rate ≥20 breathes per minute |
| Sputum colour (especially green or yellow) |

**Diagram 2:** Algorithm followed by pharmacist to support patient recruitment, clinical assessment, providing CRP testing services, and patient follow up (RA: Routine assessment) [Adapted from Sim TF, Chalmers L, Czarniak P, Hughes J, Iacob R, Lee YP, Parsons K, Parsons R, Sunderland B. Point-of-care C-reactive protein testing to support the management of respiratory tract infections in community pharmacy: a feasibility study. Research in Social and Administrative Pharmacy. 2021 Oct 1;17(10):1719-26.]


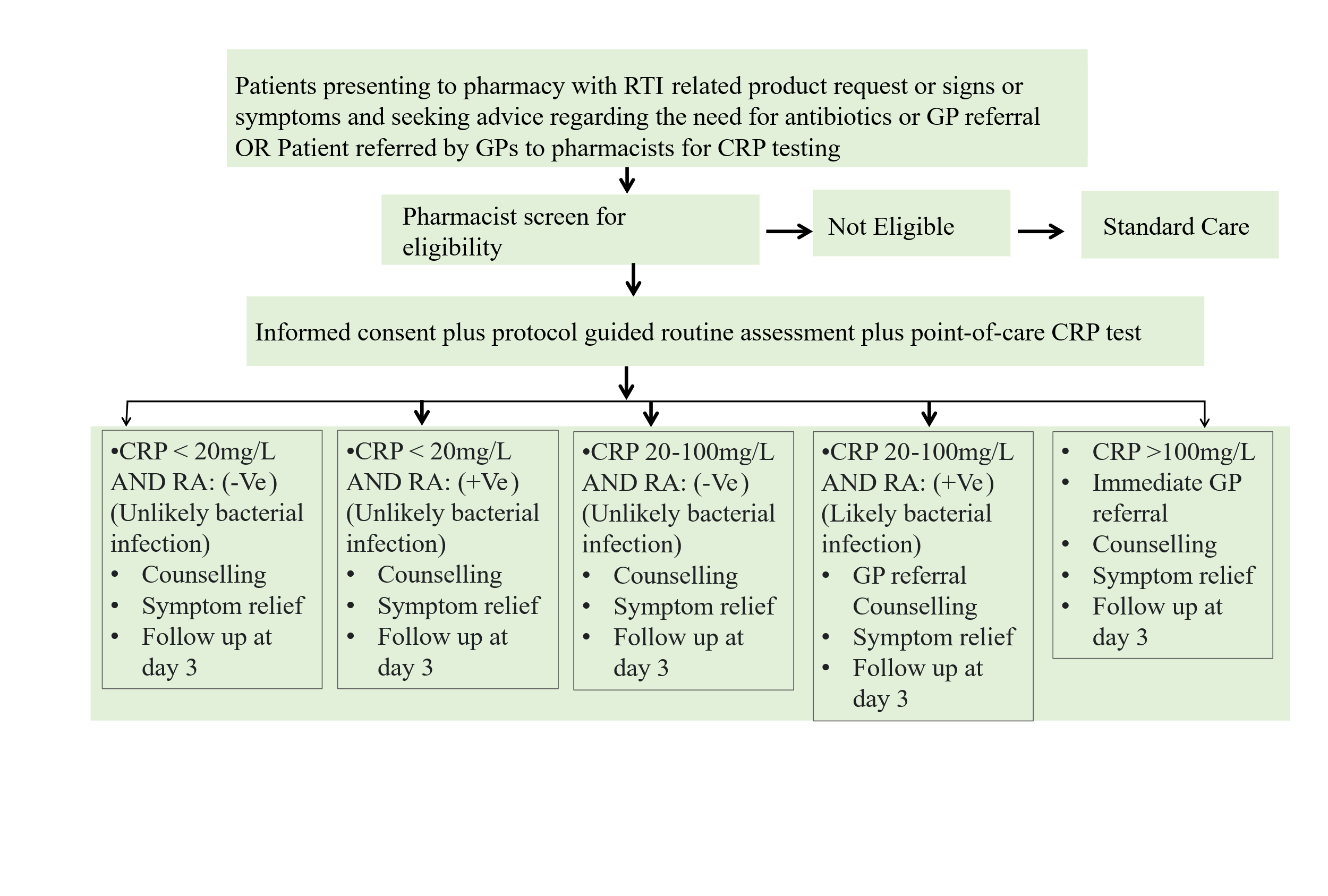


**Table 2:** Association between clinical symptoms and antibiotic prescription

| **Symptoms** | **N (%)** | **Antibiotics Prescribed**  ***N = 13*** | **No Antibiotics prescribed**  ***N = 30*** | **p-value¹** |
| --- | --- | --- | --- | --- |
| Cough | 30(70%) | 10 (77%) | 20 (67%) | 0.7 |
| Fever | 25(58%) | 11 (85%) | 14 (47%) | 0.021 |
| Congestion | 11(26%) | 2 (15%) | 9 (30%) | 0.5 |
| Sore throat | 19(44%) | 7 (54%) | 12 (40%) | 0.4 |
| Runny nose | 17(53%) | 5 (38%) | 12 (40%) | >0.9 |
| Headache | 16(37%) | 3 (23%) | 13 (43%) | 0.3 |
| Cold | 10(23%) | 3 (23%) | 7 (23%) | >0.9 |
| Asthma | 5(12%) | 3 (23%) | 2 (6.7%) | 0.2 |
| Sweating | 4(9%) | 1 (7.7%) | 3 (10%) | >0.9 |
| Phlegm | 9(21%) | 3 (23%) | 6 (20%) | >0.9 |
| Pneumonia | 3(7%) | 2 (15%) | 1 (3.3%) | 0.2 |
| Vertigo | 1(2%) | 0 (0%) | 1 (3.3%) | >0.9 |
| Vomiting | 4(9%) | 2 (15%) | 2 (6.7%) | 0.6 |
| Diarrhoea | 3(7%) | 1 (7.7%) | 2 (6.7%) | >0.9 |
| Muscle pain | 1(2%) | 0 (0%) | 1 (3.3%) | >0.9 |
| Bronchitis | 3(7%) | 1 (7.7%) | 2 (6.7%) | >0.9 |
| Sinusitis | 6(14%) | 3 (23%) | 3 (10%) | 0.3 |
| Hoarse voice | 3(7%) | 2 (15%) | 1 (3.3%) | 0.2 |
| Dizziness | 3(7%) | 0 (0%) | 3 (10%) | 0.5 |
| Sputum colour | 3(7%) | 1 (7.7%) | 2 (6.7%) | >0.9 |
| Upper Respiratory Tract Infection | 4(9%) | 1 (7.7%) | 3 (10%) | >0.9 |

¹ p-values calculated using Fisher’s exact test or Pearson’s Chi-squared test.

**Table 3:** Relation between clinical symptoms and CRP value

| **Symptoms** | **20–100+ mg/L*N = 14*** | **<20 mg/L*N = 29*** | **p-value¹** |
| --- | --- | --- | --- |
| Cough | 8 (57%) | 22 (76%) | 0.3 |
| Fever | 9 (64%) | 16 (55%) | 0.6 |
| Congestion | 3 (21%) | 8 (28%) | >0.9 |
| Sore throat | 9 (64%) | 10 (34%) | 0.065 |
| Runny nose | 5 (36%) | 12 (41%) | 0.7 |
| Headache | 5 (36%) | 11 (38%) | >0.9 |
| Cold | 3 (21%) | 7 (24%) | >0.9 |
| Asthma | 1 (7.1%) | 4 (14%) | >0.9 |
| Sweating | 0 (0%) | 4 (14%) | 0.3 |
| Phlegm | 3 (21%) | 6 (21%) | >0.9 |
| Pneumonia | 2 (14%) | 1 (3.4%) | 0.2 |
| Vertigo | 0 (0%) | 1 (3.4%) | >0.9 |
| Vomiting | 1 (7.1%) | 3 (10%) | >0.9 |
| Diarrhoea | 0 (0%) | 3 (10%) | 0.5 |
| Muscle pain | 0 (0%) | 1 (3.4%) | >0.9 |
| Bronchitis | 2 (14%) | 1 (3.4%) | 0.2 |
| Sinusitis | 2 (14%) | 4 (14%) | >0.9 |
| Hoarse voice | 1 (7.1%) | 2 (6.9%) | >0.9 |
| Dizziness | 0 (0%) | 3 (10%) | 0.5 |
| Sputum colour | 1 (7.1%) | 2 (6.9%) | >0.9 |
| Upper Respiratory Tract Infection | 1 (7.1%) | 3 (10%) | >0.9 |

¹ p-values calculated using Fisher’s exact test or Pearson’s Chi-squared test.

**Table 4:** Pharmacist’s appraisal and implementation feasibility of the CRP-PoCT program for antibiotic stewardship in respiratory infections

|  | **Set A: POCT CRP testing program** | **Is it relevant?** | **Is it affordable?** | **Can it be delivered easily?** | | **Will it be effective at reducing antimicrobial use?** | **Will it be acceptable to staff?** | **Is it safe to implement?** | **Will it improve patient care?** | **Rating of implementation feasibility out of 7 scale** |
| --- | --- | --- | --- | --- | --- | --- | --- | --- | --- | --- |
| 1 | Using POCT CRP testing service for antibiotic stewardship in RTIs | Yes | Yes | No | | Yes | No | Yes | Yes | 4 |
| 2 | Recommending delayed/back-up prescriptions based on CRP result | No | Yes | No | | Yes | No | Yes | Yes | 3 |
| 3 | Addressing patient demand for antibiotics by explaining CRP value and relevant conversation with patients | Yes | Yes | No | | Yes | No | Yes | Yes | 4 |
| 4 | Implementing POCT CRP testing program both in GP and pharmacy practices through GP-pharmacist collaboration where possible | Yes | Yes | Yes | | Yes | Yes | Yes | Yes | 5 |
| **Set B: Perceived benefits of using POCT CRP testing program (Tick)** | | | | | | | | | | |
|  | **To what extent you believe that-** | | | | **Scale** **(1 poorly believe and 5 strongly believe)** | | | | | |
| 1 | POCT CRP testing program was not time consuming | | | | 3 | | | | | |
| 2 | POCT CRP testing helped me to diagnose the severity of RTI infection? | | | | 5 | | | | | |
| 3 | POCT CRP testing result helped my decision for antibiotic therapy in RTI patients | | | | 4 | | | | | |
| 4 | POCT testing program increased my confidence for RTI management | | | | 3 | | | | | |
| 5 | POCT testing program increased my conversation with patient to address patient demand for antibiotic | | | | 5 | | | | | |
| 6 | POCT testing program increased my confidence for delayed antibiotic prescription | | | | 4 | | | | | |
| **Set C: Future implementation of POCT CRP program** | | | | | **Scale** **(1 strongly disagree and 5 strongly agree)** | | | | | |
| 1. | I would like to use POCT CRP testing in future if available in my practice | | | | 3 | | | | | |
| 2 | I would like to refer RTI-patient to a collaborated GP for doing the POCT CRP testing if available | | | | 5 | | | | | |
| 3 | POCT CRP testing program can be implemented through GP-pharmacy collaborative practice agreement | | | | 5 | | | | | |
| 4 | POCT CRP testing program can help efficient patient referral between GPs and community pharmacists if the service is available both in GP and pharmacy practices | | | | 5 | | | | | |
| 5 | Practice pharmacy technicians should support doing POCT testing in my practice | | | | 5 | | | | | |
| 6 | POCT CRP testing program in pharmacy would impact in general practice by- | | | |  | | | | | |
|  | decreasing overall antibiotic prescribing | | | | 5 | | | | | |
|  | decreasing broad spectrum antibiotic prescribing | | | | 4 | | | | | |
|  | Optimising the choice of antibiotic while prescribing | | | | 4 | | | | | |
|  | Optimising the duration of antibiotic therapy while prescribing | | | | 4 | | | | | |
| 7 | Patient Medicare should cover the costs of POCT CRP testing (e.g., $15-20) | | | | 5 | | | | | |
| 8 | POCT CRP use could increase GP-pharmacist communication with regards to RTI management (e.g., patient referral and antibiotic use) | | | | 4 | | | | | |

**Table 5: Illustrative quotes from GP and pharmacist participants on challenges and facilitators to implementing CRP-PoCT in routine practice**

| *“Clear and simple workflow protocol, no cost to the clinic and perhaps the patients could have the CRP-PoCT done prior to seeing the GPs may facilitate the testing in routine care” (GP3)* |
| --- |
| *“There were significant challenges with the testing itself. However, more to do with time and managing other pharmacy duties” (P1)* |
| *“Upskilling staff would be a priority as this would better facilitate the service” (P1)* |
| *“Medicare funding or similar, which would remunerate the pharmacy for the service and make this cost-effective for the patients” (P1)* |
